# Metabolic engineering of *Clostridium thermocellum* for *n*-butanol production from cellulose

**DOI:** 10.1186/s13068-019-1524-6

**Published:** 2019-07-23

**Authors:** Liang Tian, Peter M. Conway, Nicholas D. Cervenka, Jingxuan Cui, Marybeth Maloney, Daniel G. Olson, Lee R. Lynd

**Affiliations:** 1 Thayer School of Engineering, Dartmouth College, Hanover, NH 03755 USA; 2 Center for Bioenergy Innovation, Oak Ridge National Laboratory, Oak Ridge, TN 37830 USA; 3 Dartmouth College, Hanover, NH 03755 USA; 4 Department of Biological Sciences, Dartmouth College, Hanover, NH 03755 USA

**Keywords:** Cellulosic biofuel, *Clostridium thermocellum*, Consolidated bioprocessing, *n*-Butanol, Protein engineering

## Abstract

**Background:**

Biofuel production from plant cell walls offers the potential for sustainable and economically attractive alternatives to petroleum-based products. In particular, *Clostridium thermocellum* is a promising host for consolidated bioprocessing (CBP) because of its strong native ability to ferment cellulose.

**Results:**

We tested 12 different enzyme combinations to identify an *n*-butanol pathway with high titer and thermostability in *C. thermocellum*. The best producing strain contained the thiolase–hydroxybutyryl-CoA dehydrogenase–crotonase (Thl-Hbd-Crt) module from *Thermoanaerobacter thermosaccharolyticum*, the trans-enoyl-CoA reductase (Ter) enzyme from *Spirochaeta thermophila* and the butyraldehyde dehydrogenase and alcohol dehydrogenase (Bad-Bdh) module from *Thermoanaerobacter* sp. X514 and was able to produce 88 mg/L *n*-butanol. The key enzymes from this combination were further optimized by protein engineering. The Thl enzyme was engineered by introducing homologous mutations previously identified in *Clostridium acetobutylicum*. The Hbd and Ter enzymes were engineered for changes in cofactor specificity using the CSR-SALAD algorithm to guide the selection of mutations. The cofactor engineering of Hbd had the unexpected side effect of also increasing activity by 50-fold.

**Conclusions:**

Here we report engineering *C. thermocellum* to produce *n-*butanol. Our initial pathway designs resulted in low levels (88 mg/L) of *n-*butanol production. By engineering the protein sequence of key enzymes in the pathway, we increased the *n-*butanol titer by 2.2-fold. We further increased *n*-butanol production by adding ethanol to the growth media. By combining all these improvements, the engineered strain was able to produce 357 mg/L of *n*-butanol from cellulose within 120 h.

**Electronic supplementary material:**

The online version of this article (10.1186/s13068-019-1524-6) contains supplementary material, which is available to authorized users.

## Background

Cellulosic biofuels are widely seen as desirable and likely necessary in order to achieve a decarbonized transport sector [1]. Although cellulosic biomass is widely available at a purchase cost less than petroleum on a $/GJ basis, the high cost of processing makes current technology for biofuel production uncompetitive [2]. A new processing paradigm has recently been proposed, which has the potential for dramatic cost reduction, by combining consolidated bioprocessing (CBP) and milling during fermentation (cotreatment), termed C-CBP [3]. The thermophilic, anaerobic bacterium, *Clostridium thermocellum*, is a good candidate organism for C-CBP, because it can rapidly solubilize and ferment cellulosic biomass without pretreatment or added enzymes, the two factors responsible for the high cost of current conversion technology [4, 5]. To date, *C. thermocellum* has been engineered for CBP production of ethanol [6–8] and isobutanol [9]. However, both products are natively produced by *C. thermocellum*.

**Fig. 1:**
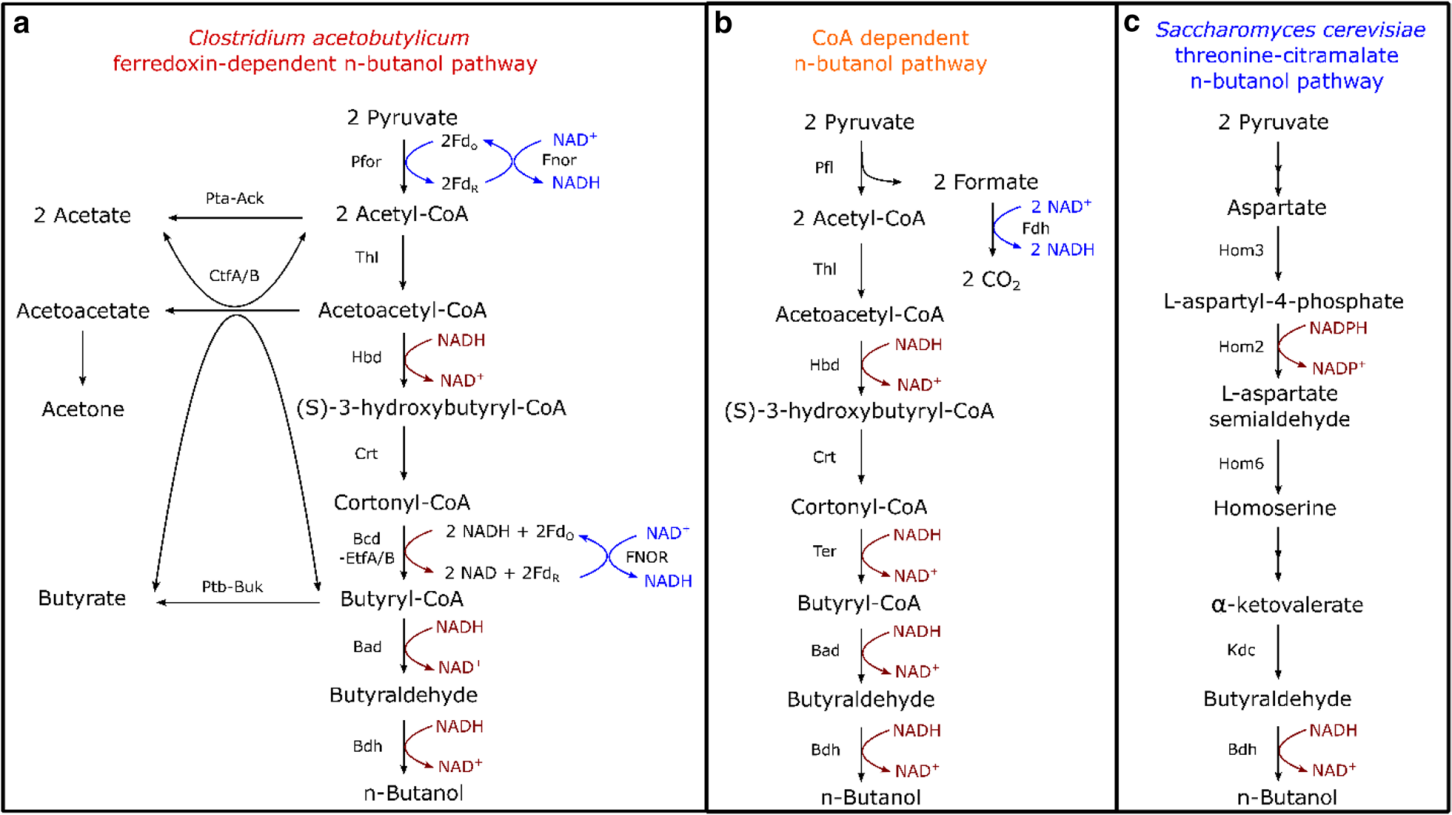
*n*-Butanol pathways summary. Pfor: pyruvate ferredoxin oxidoreductase (EC 1.2.7.1); Fnor: ferredoxin: NAD(P)^+^ oxidoreductase (EC 1.18.1.2); Pta: phosphotransacetylase (EC 2.3.1.8); Ack: acetate kinase (EC 2.7.2.1); CtfA/B: butyrate-acetoacetate CoA-transferase (EC 2.8.3.9); Ptb: phosphate butyryltransferase (EC 2.3.1.19); Buk: butyrate kinase (EC 2.7.2.7); Thl: thiolase (EC 2.3.1.9); Hbd: 3-hydroxybutyryl-CoA dehydrogenase (EC1.1.1.35); Crt: 3-hydroxybutyryl-CoA dehydratase (EC 4.2.1.55); Bcd/Etf: butyryl-CoA dehydrogenase/electron transfer protein; Ter: trans-2-enoyl-CoA reductase (EC 1.3.1.44); Bad: butyraldehyde dehydrogenase (EC 1.2.1.57); Bdh: alcohol dehydrogenase (EC 1.1.1.1); Pfl: pyruvate formate-lyase (EC2.3.1.54); Hom3: aspartate kinase (AK) gene; Hom2: Aspartic beta semi-aldehyde dehydrogenase; Hom6: homoserine dehydrogenase (HSDH) gene Kdc, 2-keto-acid decarboxylases

*n*-Butanol is an “advanced biofuel” with a higher energy content and lower volatility compared to ethanol. In addition to its potential use as a biofuel, it is also widely used as a solvent in the chemical industry [10]. The most well-known biological process for *n*-butanol production is the acetone–butanol–ethanol (ABE) fermentation using *Clostridia* species (Fig. 1a) [11, 12]. For a long time, lack of genetic tools and low alcohol tolerance (20 g/L for engineered strains) have limited the development of this process [13]. However, a recent study combining metabolic engineering and process engineering demonstrated the potential of commercial-level production of *n*-butanol by *Clostridium acetobutylicum* [12]. Various attempts have been made to transfer the *Clostridial n*-butanol pathway to more suitable industrial organisms including *Escherichia coli* and *Saccharomyces cerevisiae*. A common feature of these approaches is that they eliminate ferredoxin-linked enzymes, such as butyryl-CoA dehydrogenase/electron transfer protein (Bcd/EtfAB) and ferredoxin: NAD(P)^+^ oxidoreductase (Fnor), and use the Ter enzyme instead (Fig. 1b). This CoA-dependent pathway has allowed high levels (up to 30 g/L) of *n-*butanol production in *E. coli* [14, 15]. However, putting this pathway in *S. cerevisiae* has been less successful, with a maximum titer of only 100 mg/L [16]. A third option for *n*-butanol production involves using the threonine biosynthesis pathway and/or the citramalate pathway to produce alpha-ketobutyrate, followed by decarboxylation and reduction to *n*-butanol [17] (Fig. 1c). Introducing this pathway into *S. cerevisiae* has allowed production of 835 mg/L *n*-butanol. In addition to these three pathways, which allow conversion of sugar to *n*-butanol, some organisms have a native ability to convert butyrate to *n*-butanol, and these organisms may be a source of enzymes for *n*-butanol production [18, 19].

Cellulosic *n*-butanol production was first achieved by mesophilic cellulosic *Clostridium* species [20, 21]. However, the low efficiency of cellulose solubilization limits the application of these mesophilic cellulosic organisms (e.g., *Clostridium cellulovorans* needs 10 days to consume 7 g/L Avicel) [20, 22, 23]. A recent study reported the production of *n-*butanol from cellulose using a newly isolated *Thermoanaerobacterium* species [24]. However, the limited extent of conversion (only 33% of the initial cellulose was consumed) and slow rate (10 g/L cellulose consumed in 18 days) make this strain less desirable for commercial application. Furthermore, the lack of essential cellulolytic genes from the carbohydrate-active enzyme database (CAZy) categories of GH6, GH7, GH9, GH12 and GH48 suggests that this strain may not be truly cellulolytic [25–27].

An important first step in engineering *C. thermocellum* to produce *n-*butanol is identifying a thermostable *n-*butanol pathway. So far, there are two thermophilic *n-*butanol pathways published, and based on these studies, *n-*butanol pathway genes from thermophilic organisms *Thermoanaerobacterium thermosaccharolyticum* [28], *Thermoanaerobacter* sp. X514 and *Spirochaeta thermophila* [29] were selected. In addition, some other key genes from the mesophilic species *Clostridium acetobutylicum* [30, 31], *Cupriavidus necator* and *Aeromonas caviae* were also selected [15]. After testing several different combinations, the best performing pathway was further optimized by protein engineering of key enzymes.

## Results

### Pathway combinations

**Fig. 2:**
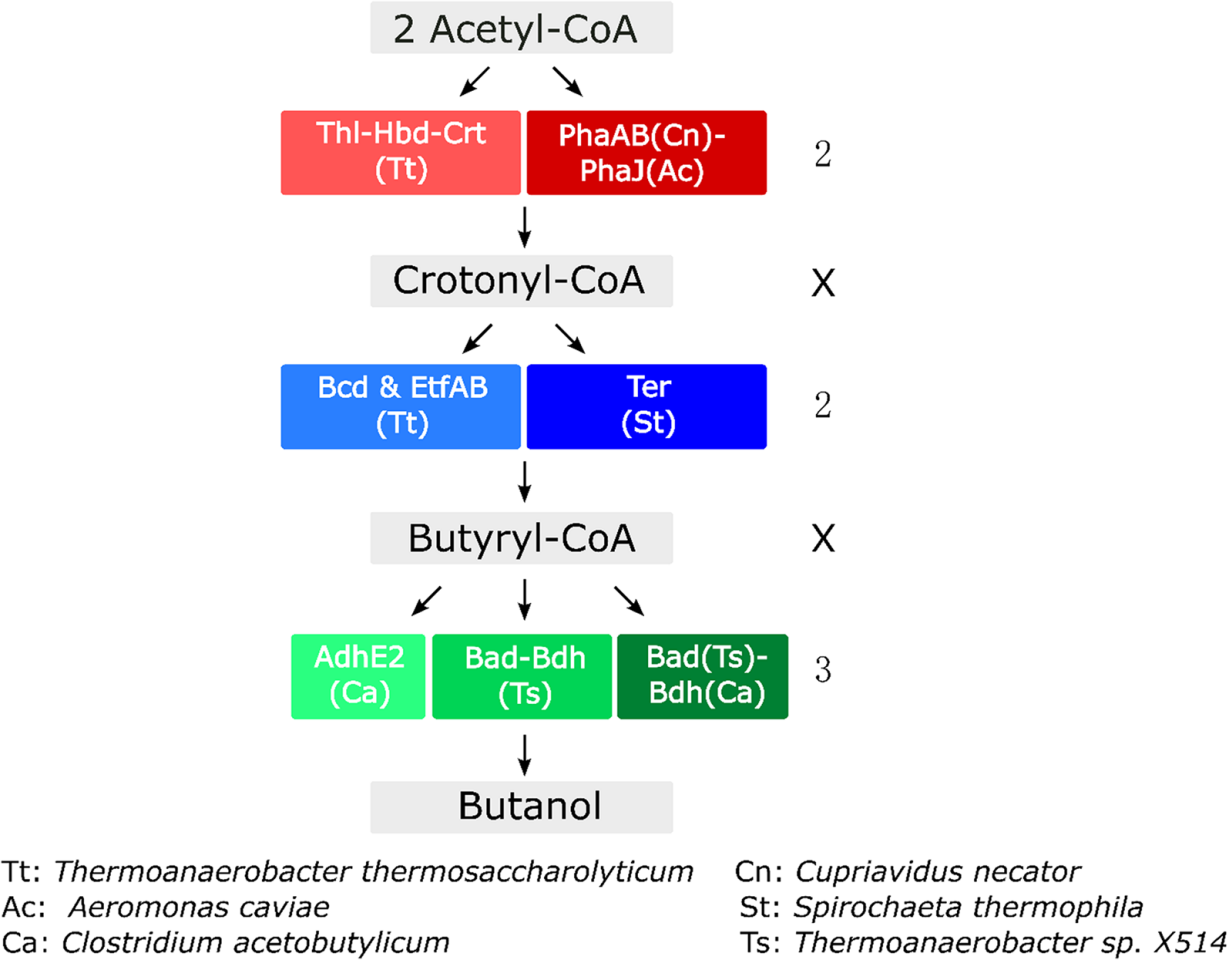
*n*-Butanol pathway combinations. Thl: thiolase (EC 2.3.1.9); Hbd: 3-hydroxybutyryl-CoA dehydrogenase (EC1.1.1.35); Crt: 3-hydroxybutyryl-CoA dehydratase (EC 4.2.1.55); PhaA: acetoacetyl-CoA thiolase/synthase; PhaB: 3-hydroxybutyryl-CoA dehydrogenase; PhaJ: PHA synthase; Bcd/Etf: butyryl-CoA dehydrogenase/electron transfer protein; Ter: trans-2-enoyl-CoA reductase (EC 1.3.1.44); Bad: butyraldehyde dehydrogenase (EC 1.2.1.57); Bdh: alcohol dehydrogenase (EC 1.1.1.1); AdhE2: bifunctional acetaldehyde dehydrogenase/alcohol dehydrogenase

To find the best combination of pathway enzymes for thermophilic *n-*butanol production in *C. thermocellum*, we tested pathway genes from several different species and engineered strains. In total, 12 different combinations were constructed on plasmids (Fig. 2) and the native *C. thermocellum* promoter from gene Clo1313_2638 [32] was used to drive expression. *C. thermocellum* (strain LL1299) was transformed with each of the plasmids to test *n-*butanol production. All strains were cultured in MTC-5 medium for 7 days before analysis.

Based on the final *n-*butanol titer, the BT05 pathway (*thl*-*hbd*-*crt*-*ter*-*bad*-*bdh*) was selected for further optimization (Table 1). The genes *Ts_bad* and *Ts_bdh* were integrated downstream of the Clo1313_2637 gene and driven by the *C. thermocellum* enolase promoter [32]. The genes *Tt_thl* and *Tt_hbd* were integrated upstream of gene Clo1313_2638 and driven by the Clo1313_2638 promoter. These two integration loci have been previously shown to support high levels of gene expression [7]. The *Tt_crt* and *St_ter* genes were integrated at the lactate dehydrogenase (*ldh*) locus (Clo1313_1160) with the concurrent deletion of *ldh* [33] and also driven by the *C. thermocellum* enolase promoter [32]. To further increase *n-*butanol titer, the Clo1313_1353-1356 genes responsible for isobutanol production were deleted [7]. The final strain was named LL1669. Genome modifications were confirmed by whole-genome sequencing. Then, strain LL1669 was cultured in serum bottles in MTC-5 medium with 20 g/L cellobiose, which is the same set of conditions that was used for the results in Table 1. Compared to plasmid-based expression (Table 1, BT05), the *n-*butanol titer for the chromosomally expressed pathway decreased from 87 to 42 mg/L (Table 4). This may be due to differences in gene copy number (single copy on the genome vs. multiple copies when expressed from a plasmid).

**Table 1:** *n*-Butanol production of different enzyme combinations

Combination number	*n*-Butanol pathway			*n-*Butanol (mg/L)	Ethanol (mg/L)	Acetate (mg/L)	Lactate (mg/L)
BT01	Thl-Hbd-Crt	Bcd-EtfAB	AdhE2	10 ± 2^a^	3100 ± 100	2800 ± 100	1500 ± 200
BT02			Bad-Bdh	75 ± 6	2600 ± 100	2400 ± 100	1400 ± 200
BT03			Bad-Bdh(Ca)	15 ± 1	3200 ± 200	3000 ± 100	1500 ± 300
BT04		Ter	AdhE2	13 ± 1	3100 ± 200	2900 ± 200	1200 ± 200
BT05			Bad-Bdh	87 ± 8	2500 ± 200	2200 ± 100	1100 ± 100
BT06			Bad-Bdh(Ca)	20 ± 2	3100 ± 200	2700 ± 100	1200 ± 200
BT07	PhaAB-PhaJ	Bcd-EtfAB	AdhE2	5 ± 1	2900 ± 100	3200 ± 200	1300 ± 200
BT08			Bad-Bdh	54 ± 3	2900 ± 200	3000 ± 100	1200 ± 100
BT09			Bad-Bdh(Ca)	10 ± 2	3100 ± 100	2800 ± 100	1200 ± 100
BT10		Ter	AdhE2	6 ± 1	3100 ± 100	2900 ± 200	1300 ± 300
BT11			Bad-Bdh	41 ± 5	2900 ± 200	2600 ± 100	1600 ± 200
BT12			Bad-Bdh(Ca)	8 ± 1	3100 ± 200	3000 ± 100	1500 ± 100

^a^Error represents one standard deviation, *n* = 3

### *n-*Butanol pathway enzyme characterization

**Fig. 3:**
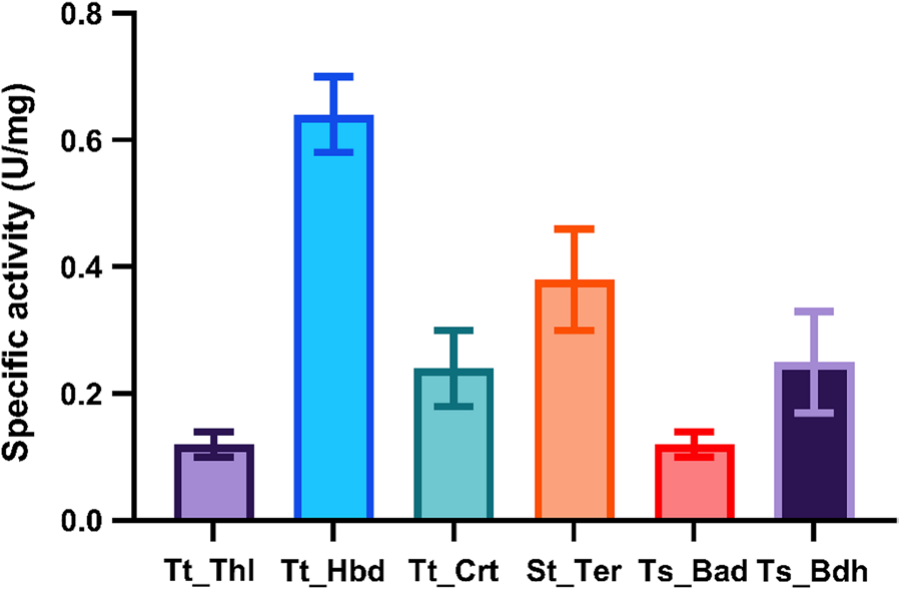
Specific activities of the *n*-butanol pathway enzymes in *Clostridium thermocellum.* Cell-free extract was used for this experiment. The individual specific activities (μmol NADH oxidation/min/mg) were measured by the addition of acetyl-CoA for Thl, acetoacetyl-CoA for Hbd, 3-hydroxybutyryl-CoA for Crt, crotonyl-CoA for Ter, butyryl-CoA for Bad and butyraldehyde for Bdh. Error represents one standard deviation, *n* = 3

To identify which enzymes are most likely limiting overall flux, the activities of all the enzymes of the BT05 *n-*butanol pathway in *C. thermocellum* LL1669 cell-free extract were measured (Fig. 3). The activities of Thl and Bad were the lowest among all the enzymes in the pathway which indicated that one or both of them might limit flux.

At the same time, all the enzymes were also expressed and purified from *E. coli*. Their kinetic parameters were measured (Table 2). Looking at both *K*_m_ and catalytic efficiency (*k*_cat_/*K*_m_), Thl and Bdh again seem likely to limit flux (due to high *K*_m_ and low catalytic efficiency). Since Bdh can also convert acetaldehyde to ethanol, any improvement in activity might also lead to an increase in (undesired) ethanol production. Therefore, Thl was selected as the first target for protein engineering.

**Table 2:** Kinetic parameters of all the enzymes in the *n-*butanol pathway

Enzyme	Substrate for *K*_m_ measurements	*K*_m_ (mM)	*V*_max_ (µmol/min/mg)^a^	*k*_cat_/*K*_m_ (10^3^ M^−1^s^−1^)
Tt_Thl	Acetyl-CoA	2.4 ± 0.58^b^	19.2 ± 1.8	11.7 ± 3.5
Tt_Hbd	Acetoacetyl-CoA	0.03 ± 0.01	67.4 ± 7.4	3955 ± 658
Tt_Crt	Hydroxybutyryl-CoA	0.6 ± 0.1	181.3 ± 15.4	607 ± 54
St_Ter	Crotonyl-CoA	0.03 ± 0.01	22.6 ± 2.2	924 ± 89
Ts_Bad	Butyryl-CoA	0.2 ± 0.1	16.1 ± 2.2	79.1 ± 1.5
Ts_Bdh	Butyraldehyde	4.2 ± 0.9	27.2 ± 3.4	9.1 ± 0.9
Ts_Bad	Acetyl-CoA	0.09 ± 0.01	5.2 ± 0.7	71.1 ± 6.9
Ts_Bdh	Acetaldehyde	5.8 ± 0.8	31.2 ± 3.1	7.6 ± 0.3

^a^0.3 mM NADH was used as cofactor

^b^Error represents one standard deviation, *n* = 3

### Tt_Thl protein engineering

There are two studies that describe protein engineering to increase the performance of the *Clostridium acetobutylicum* thiolase enzyme (Ca_Thl). In one study, the redox switch regulation of Ca_Thl was disrupted by three amino acid substitutions (V77Q, N153Y and A286K). The resulting variant enzyme exhibited higher activity, and butanol titer increased from 4.5 to 7.4 g/L [34]. In a second study, variant enzymes were screened for increased resistance to CoA inhibition. A variant with three amino acid substitutions (R133G, H156N and G222V) was identified which increased *n-*butanol titer by 18% [35]. To map the Ca_Thl mutations onto our Tt_Thl protein, a homology structure model of Tt_Thl was constructed using the crystal structure of Ca_Thl (PDB: 4WYR), since these two protein sequences share 51% similarity. Mutations corresponding to the Ca_Thl variant in Kim et al. [34] (V77Q, N153Y and S287K) were transferred to Tt_Thl. This variant was named M1. Mutations corresponding to the Ca_Thl variant in Mann et al. (R133G, H156N, G222V) were separately transferred to Tt_Thl (R133G, H156N, P222F and N223V). The substitution G222V of Ca_Thl corresponds to the substitution N223V from Tt_Thl. The adjacent of residue P222 of Tt_Thl was also changed to its corresponding residue F221 from Ca_Thl. This variant was named M2. Both variant enzymes were purified from *E. coli,* and kinetic parameters were measured (Table 3). Compared to the wild-type Tt_Thl, Tt_Thl M1 variant had higher *K*_m_ and lower *V*_max_ values. The Tt_Thl M2 variant had a similar *K*_m_ compared to the wild type; however, its *V*_max_ was two times higher. In addition, Tt_Thl M2 was less sensitive toward to its physiological inhibitor Coenzyme A (Additional file 1: Figure S1).

**Table 3:** Kinetic parameters of enzyme Tt_Thl and the variants

Enzyme	*K*_m_ (mM) (acetyl-CoA as substrate)	*V*_max_ (µmol/min/mg)
Thl	2.4 ± 0.6^a^	19.2 ± 3.2
Thl M1	3.3 ± 0.7	15.3 ± 4.3
Thl M2	2.6 ± 0.5	45.2 ± 5.2

^a^Error represents one standard deviation, *n* = 3

To test the effect of variant Tt_Thl on butanol production, both the original Tt_Thl and the variant Tt_Thl M2 were overexpressed in strain LL1669 (Table 4). Overexpression of the original Tt_Thl increased *n-*butanol titer from 42 to 58 mg/mL. The strain with Tt_Thl M2 increased *n-*butanol titer a further 19% to 69 mg/mL.

**Table 4:** *n*-Butanol production of different strains

Strain	Plasmid	*n*-Butanol titer (mg/L)^a^
LL1669	Empty vector control	42 ± 4
	pLT_207 (*Tt_thl*)	58 ± 7
	pLT_208 (*Tt_thl M2*)	69 ± 8
	pLT_228 (*Tt_thl M2, Tt_hbd, St_ter*)	89 ± 9
	pLT_229 (*Tt_thl M2, Tt_hbd M3, St_ter M*)	195 ± 12

^a^ Error represents one standard deviation, *n* = 3, for all pairs of results, *p* ≤ 0.05

### Cofactor preference optimizations

**Fig. 4:**
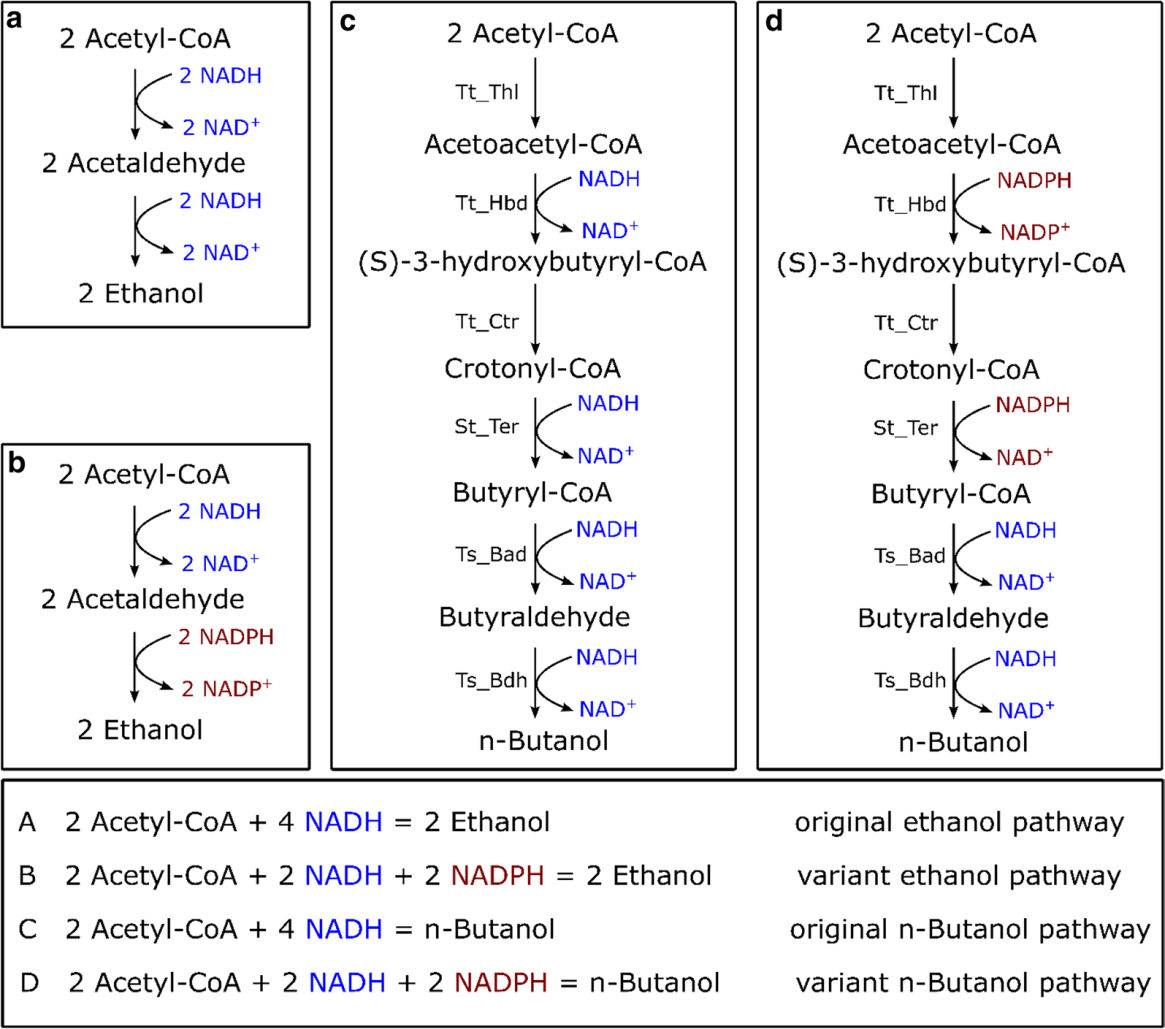
Ethanol and *n*-butanol pathways comparison. **a**, **b** The cofactor specificity of the original ethanol pathway and variant ethanol pathway, respectively; **c**, **d** the cofactor specificity of the original *n*-butanol pathway and the variant *n*-butanol pathway, respectively

Batch fermentations resulted in the formation of ethanol, acetate and lactate, in addition to *n-*butanol (Table 1). Although we have previously been able to delete the native alcohol dehydrogenase, *adhE* in wild-type *C. thermocellum* [36], deletion of *adhE* in strain LL1669 was unsuccessful. Previous studies have demonstrated that changing the cofactor preference from NADH to NADPH can increase ethanol production and tolerance in *C. thermocellum* [37, 38]. In Fig. 4, we show the cofactor specificities of the ethanol and *n-*butanol pathways. We hypothesized that change in the cofactor preference of the *n-*butanol pathway would also increase the flux from acetyl-CoA to the *n-*butanol pathway and away from the ethanol pathway (Fig. 4c, d).

**Fig. 5:**
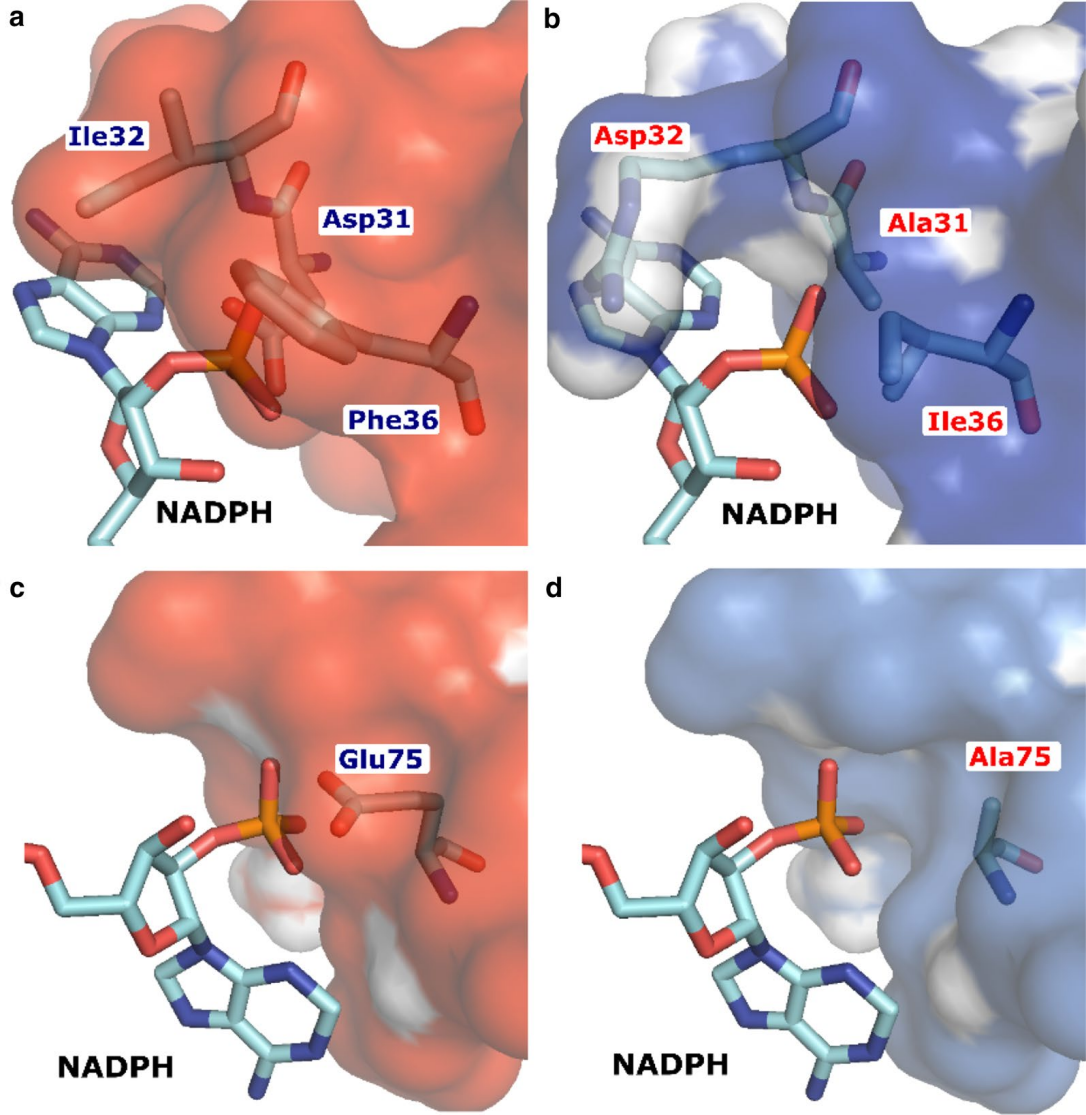
Homology modeling and docking analysis of the phosphate in NADPH interacting with **a** Tt_Hbd; **b** Tt_Hbd(D31A I32R P36I); **c** St_Ter; and **d** St_Ter (E75A)

Tt_Hbd and St_Ter were selected for engineering, and the CSR-SALAD online tool was used to design a cofactor specificity reversal library [39]. Details of the library construction and variant screening are described in the Methods section. To understand the impact of mutations on cofactor specificity, we performed homology modeling and docking (Fig. 5). The crystal structure of 3-hydroxybutyryl-CoA dehydrogenase from *Clostridium butyricum* (4KUG) and the crystal structure of trans-2-enoyl-CoA reductase from *Treponema denticola* (4FBG) [38] were used as templates due to their high level of homology to our protein sequences.

The amino acid residue D31 of Tt_Hbd and E75 of St_Ter may interfere with the 2′-phosphate group of NADPH because of the electrostatic repulsion (both are negatively charged) and steric hindrance. With the substitutions of alanine, the 2′-phosphate group of NADPH can access the binding pocket. In addition, the substitution of I32R in Tt_Hbd, which carries a positive charge, can interact with the 2′-phosphate group and increase stability.

Interestingly, although we did not set out to engineer the *V*_max_ of the two enzymes, we found that several mutations increased *V*_max_ for both Tt_Hbd and St_Ter. For Tt_Hbd, the variant with the substitutions of D31A, I32R and P36I, *V*_max_ was increased by 50-fold with NADPH as the cofactor (Table 5). For St_Ter, the variant with the substitution of E75A, *V*_max_ was increased by fivefold (Table 5). No significant change in *V*_max_ was found for mutations at other sites. It is also worth mentioning that for all variants, the *V*_max_ of the Tt_Hbd and St_Ter enzymes decreased when NADH was used as cofactor. To test the effect of cofactor specificity reversal, the both the original and variant genes (plasmids pLT_228 and pLT_229, respectively) were overexpressed in strain LL1669. The strain carrying the variant genes (LL1668) produced 195 ± 12 mg/L *n-*butanol which was 2.2 times higher than the strain carrying the original genes (Table 4).

**Table 5:** Kinetic parameters of enzymes Tt_Hbd and St_Ter and their variants, purified from *E. coli*

Enzyme	NADH		NADPH	
	*K*_m_ (mM)	*V*_max_ (µmol/min/mg)	*K*_m_ (mM)	*V*_max_ (µmol/min/mg)
Hbd^a^	0.05 ± 0.02^c^	61.4 ± 7.2	1.1 ± 0.2^a^	15.2 ± 2.5
Hbd (D31A)	0.06 ± 0.02	38.5 ± 4.5	0.03 ± 0.01	485.3 ± 34.2
Hbd (I32R)	0.08 ± 0.02	25.1 ± 6.3	0.04 ± 0.02	152.1 ± 12.2
Hbd (P36I)	0.08 ± 0.01	24.2 ± 2.1	0.04 ± 0.01	128.2 ± 15.3
Hbd M3 (D31A I32R P36I)	0.11 ± 0.02	26.6 ± 3.7	0.03 ± 0.01	764.9 ± 35.7
Ter^b^	0.03 ± 0.01	18.2 ± 2.3	0.4 ± 0.1	12.2 ± 1.5
Ter M (E75A)	0.6 ± 0.1	10.3 ± 1.5	0.02 ± 0.01	68.1 ± 6.5

^a^Acetoacetyl-CoA was substrate of Hbd assay

^b^Crotonyl-CoA was the substrate of Ter assay

^c^Error represents one standard deviation, *n* = 3

### Cellulose fermentation

**Fig. 6:**
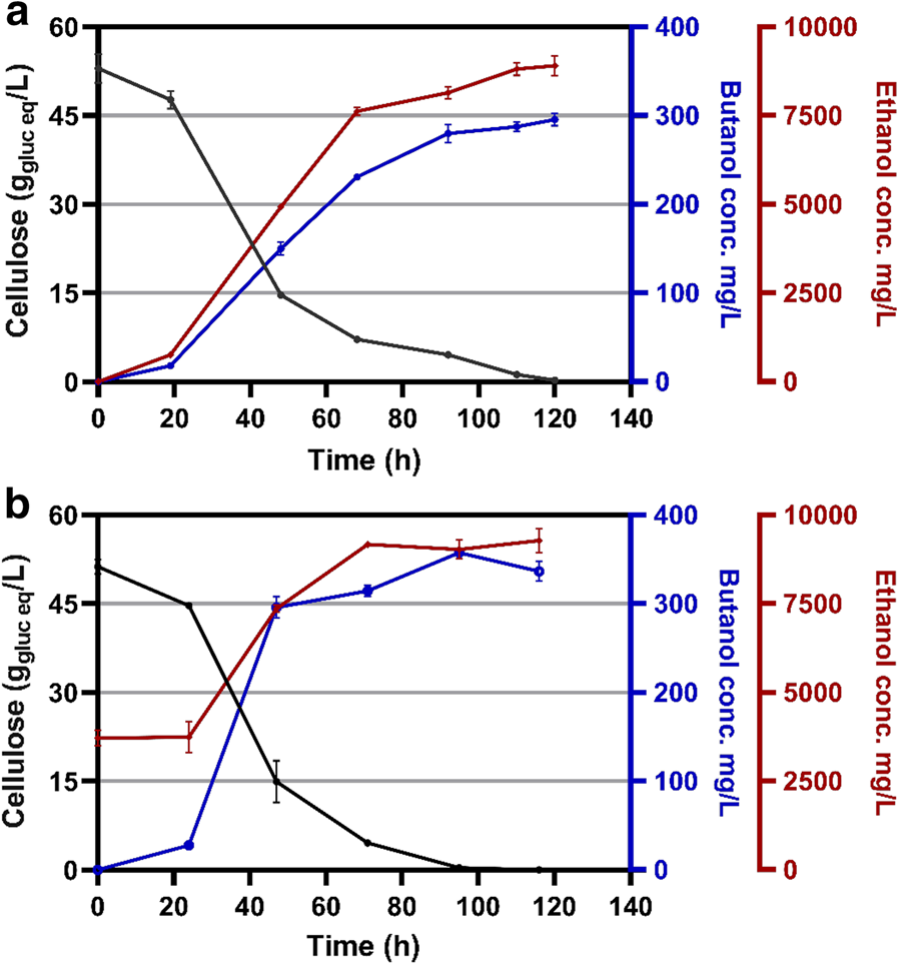
Fermentation profile of strain LL1668 in a bioreactor. The strain was grown on minimal medium (MTC-5) in a bioreactor with pH regulation in the absence (**a**) or presence (**b**) of 4 g/L added ethanol. Error bars represent one standard deviation, *n *= 3

To evaluate *n-*butanol production under conditions slightly closer to the industrial practice, we performed batch fermentations of strain LL1668 (LL1669 with the addition of the variants *thlM2*, *hbdM* and *terM* genes, see Tables 6 and 7 for a complete description) in pH-controlled bioreactors with 50 g/L (Avicel PH105). Over the course of a 5-day fermentation, 95% of the substrate was consumed (Fig. 6a) and the final *n-*butanol titer was 295 ± 5 mg/L. The main by-products were ethanol with a titer of 8750 ± 80 mg/L and acetate with a titer of 4880 ± 50 mg/L. The other by-products, including pyruvate, lactate and formate, were all less than 500 mg/L. To attempt to reduce net flux to ethanol, we added ethanol to the culture medium before inoculation (Fig. 6b). We chose a concentration of 4000 mg/L because this is higher than the amount of ethanol produced during our initial fermentation experiments (Table 1). In these conditions, the *n-*butanol titer increased by 20% and the final titer was 357 ± 3 mg/L.

**Table 6:** Strain used in this work

Organism	Strain	Description	Accession number^a^	Source or reference
*C. thermocellum*	LL1004	Wild-type *C. thermocellum* strain DSM 1313	CP002416	DSMZ
	AG929	DSM1313 Δhpt Δ*Clo1313_0478*	SRP097241	[8]
	LL1644	Strain AG929 Δ*ldh* with gene *Tt_thl,Tt_hbd, Tt_crt, St_ter,Ts_bad* and *Ts_bdh* integrated in the genome	SRP190757	This work
	LL1669	Strain LL1644 Δ*Clo1313_1353*-*1356*	SRP190758	This work
	LL1668	LL1669 with pLT_229	SRP190756	This work
*E. coli*	T7 Express lysY/lq	Used for heterologous protein expression		New England Biolabs
	DH5α	Used for plasmid screening and propagation		New England Biolabs

^a^For strains with sequenced genomes, this is the GenBank accession number. For re-sequenced strains, this is the Sequence Read Archive (SRA, https://www.ncbi.nlm.nih.gov/sra) accession number

**Table 7:** Plasmids used in this work

Plasmid	Description^a^	GenBank accession number
pDGO143	Gene expression plasmid for *C. thermocellum* [8]	KX259110
BT01	pDGO143 with *Tt_thl, Tt_hbd, Tt_crt, Ts_ bcd, Ts_etfAB and Ca_adhE2*	MK524015
BT02	pDGO143 with *Tt_thl, Tt_hbd, Tt_crt, Ts_ bcd, Ts_etfAB, Ts_bad and Ts_bdh*	MK524016
BT03	pDGO143 with *Tt_thl, Tt_hbd, Tt_crt, Ts_ bcd, Ts_etfAB, Ts_bad and Ca_bdh*	MK542521
BT04	pDGO143 with *Tt_thl, Tt_hbd, Tt_crt, St_ter and Ca_adhE2*	MK542522
BT05	pDGO143 with *Tt_thl, Tt_hbd, Tt_crt, St_ter, Ts_bad and Ts_bdh*	MK542523
BT06	pDGO143 with *Tt_thl, Tt_hbd, Tt_crt, St_ter, Ts_bad and Ca_bdh*	MK542524
BT07	pDGO143 with *Re_phaAB, Ac_phaJ, Ts_ bcd, Ts_etfAB and Ca_adhE2*	MK542525
BT08	pDGO143 with *Re_phaAB, Ac_phaJ, Ts_ bcd, Ts_etfAB, Ts_bad and Ts_bdh*	MK542526
BT09	pDGO143 with *Re_phaAB, Ac_phaJ, Ts_ bcd, Ts_etfAB, Ts_bad and Ca_bdh*	MK542527
BT10	pDGO143 with *Re_phaAB, Ac_phaJ, St_ter and Ca_adhE2*	MK542528
BT11	pDGO143 with *Re_phaAB, Ac_phaJ, St_ter, Ts_bad and Ts_bdh*	MK542529
BT12	pDGO143 with *Re_phaAB, Ac_phaJ, St_ter, Ts_bad and Ca_bdh*	MK542530
pD861-CH	*E. coli* rhamnose-inducible expression vectors from ATUM (Newark, CA)	
pLT_181	pD861-CH with *Tt_thl* gene	MK542531
pLT_182	pD861-CH with *Tt_thlM1* gene	MK542532
pLT_183	pD861-CH with *Tt_thlM2* gene	MK542533
pLT_190	pD861-CH with *Tt_hbd* gene	MK542534
pLT_193	pD861-CH with *Tt_crt* gene	MK542535
pLT_194	pD861-CH with *St_ter* gene	MK542536
pLT_195	*pD861*-*CH with Ts_bad* gene	MK542537
pLT_196	*pD861*-*CH with Ts_bdh* gene	MK542538
pLT_164	Integrate *Ts_bad* and *Ts_bdh* to *C. thermocellum* genome *C. thermocellum* enolase promoter was used for gene expression [32]	MK542539
pLT_191	Integrate *Tt_thl* and *Tt_hbd* to *C. thermocellum* genome *C. thermocellum* Clo1313_2638 promoter was used for gene expression [32]	MK542540
pLT_199	Integrate *Tt_crt* and *St_ter* to *C. thermocellum* genome *C. thermocellum* enolase promoter was used for gene expression [32]	MK542541
pJGW37	Gene expression plasmid for *C. thermocellum*	[46]
pLT_208	pJGW37 with *Tt_thlM1*	MK542542
pLT_209	pJGW37 with *Tt_thlM2*	MK542543
pLT_228	pJGW37 with *Tt_thlM2, Tt_hbd* and *St_ter*	MK542544
pLT_229	pJGW37 with *Tt_thlM2, Tt_hbdM* and *St_terM*	MK542545

^a^Genes are listed in the 5′–3′ direction in their operon

## Discussion

### Effect of gene choice

We have demonstrated that a variety of combinations of *n-*butanol pathway enzymes can work in *C. thermocellum*. There are several factors that may influence the performance of the enzymes, including gene expression level, plasmid stability and enzyme stability. To mitigate the influence of these factors, the same promoter and plasmid were used for gene expression. RBS sequences were individually designed for each gene, in an attempt to even out the translation initiation rate [40]. For acetyl-CoA conversion to crotonyl-CoA, the Thl-Hbd-Crt enzymes work about twofold better than PhaAB and PhaJ. This is similar to the results shown by Bond-Watts et al. in *E. coli* [41]). For crotonyl-CoA conversion to butyryl-CoA, Ter works about 10% better than Bcd-EtfAB which was also found in a previous study [14]. Many other groups studying *n-*butanol production have come to a similar conclusion. For example, Shen et al. found an 18-fold improvement when comparing Bcd-EtfAB to Ter [14]. One possible explanation for the poor performance of Bcd-EtfAB is that it does not interact well with native *C. thermocellum* ferredoxin; however, we did not explicitly test this. For butyryl-CoA to butanol conversion, Bad-Bdh works 8-fold better than AdhE2 and fivefold better than Bad-Bdh (Ca). By engineering the enzyme responsible for the first committed step in the *n-*butanol pathway (i.e., Thl) and changing the cofactor specificity of Hbd and Ter, *n-*butanol titer was increased by twofold (from 89 to 195 mg/mL). All the enzymes from the optimal combinations are from thermophilic organisms, and this thermostability may help them function appropriately in *C. thermocellum*.

### Butanol titer limitations

Wild-type *C. thermocellum* can tolerate 5 g/L *n-*butanol and with adaptation, up to 15 g/L [37]. Since this is 14- and 42-fold higher than the titers reported in this work, it suggests that *n-*butanol tolerance is not likely a limiting factor. A more likely limitation is the step converting acetyl-CoA to acetoacetyl-CoA (i.e., thiolase). This reaction has a large positive Gibbs free energy (Δ_r_G’°) of 26 kJ/mol [42], and thus, the reverse reaction is strongly favored. In order for this reaction to support high flux, it requires a high ratio of acetyl-CoA to acetoacetyl-CoA (about 15,000:1). Since even under ideal circumstances, this enzyme will be working near equilibrium, it should also have high activity. In this work, we report a specific activity of about 0.1 U/mg CFE for Thl, which is lower than that reported by Bhandiwad et al. (5 U, for a strain that produced 1 g/L butanol [28] and also lower than that reported for AtoB (10-17 U [14]). In fact, all of the enzyme activities reported here (except Bad and Bdh) have lower values than those reported by Shen et al. (Hbd: 2.7–4.6 U; Crt: 97.9–128.4 U; Ter: 1.2–3.7 U; Bad: 0.014 U; and Bdh: 0.007 U), suggesting that enzyme production may be the primary limitation to *n-*butanol titer in *C. thermocellum*. The high *K*_m_ value of the thiolase might be another limiting factor. Without a sufficiently large acetyl-CoA pool or an efficient product trap, there is no driving force for the formation of acetoacetyl-CoA. In a previous study, AtoB from *E.coli* was demonstrated to have a better performance than Thl [43], but AtoB was not tested in this study since it was from a mesophilic organism, which might lead to problems with thermostability.

Another possible limitation to butanol production is competition with ethanol production for reducing equivalents (NADH, NADPH). In *C. thermocellum*, there seems to be a natural excess of NADPH [44], and we attempted to take advantage of this by changing the cofactor specificities of the Hbd and Ter enzymes from NADH to NADPH. However, it might be possible, through additional engineering, to further improve the driving force of NADPH production. Furthermore, our engineered pathway (Fig. 4d) still uses both NADH and NADPH. Modifying the two remaining NADH-linked reactions to use NADPH would simplify engineering by eliminating the need to independently balance the driving forces for both cofactors. *C. thermocellum* uses two different enzymes to transfer electrons from ferredoxin to NAD(P)^+^, NfnAB and Rnf [45]. If all of the reduction steps of the butanol pathway were NADPH linked, deleting the *rnf* operon might further increase butanol production.

## Conclusions

*n-*Butanol can be produced by a variety of microorganisms, using native or non-native pathways. However, to date there have been no reports of *n-*butanol production by a thermophilic organism using crystalline cellulose as the only substrate. Here, we engineered *C. thermocellum*, one of the most efficient cellulosic bacteria, to produce *n-*butanol. Overall, the strategies used here are not specific for *n-*butanol production and can be explored for other products. This study advances the understanding of how thermophilic cellulosic organisms such as *C. thermocellum* can be used to produce non-native products.

## Methods

### Bacterial strains, plasmids, media and cultivation

Strains and plasmids used in this study are listed in Tables 6 and 7. The plasmid pDGO143 was used as the backbone for heterologous genes expression in *C. thermocellum.* This plasmid has both the p15A origin for replication in *E.coli* and the *repB* origin for replication in *C. thermocellum* [8]. For protein purification in *E. coli*, the rhamnose-inducible expression vector pD861-CH from ATUM (Newark, CA) was used as the backbone. The plasmid pSH106 was used as the backbone for gene integration in *C. thermocellum* [7]. The plasmid pJGW37 was also used as the backbone for the gene expression in *C. thermocellum,* since it has a different origin of replication than pDGO143 [46]. The *C. thermocellum* native promoter from gene Clo1313_2638 [32] was used for plasmids BT01–BT12, and *Thermoanaerobacterium saccharolyticum pforA* promoter was used for plasmids pLT_208, 209, 228 and 229. Additional details of plasmid design are given in Table 7. RBS sequences of each gene were designed using an online RBS calculator tool [40]. Plasmids were constructed via isothermal assembly [47] using a commercial kit sold by New England Biolabs (NEBuilder^®^ HiFi DNA Assembly Master Mix, catalog number E2621). The DNA purification of plasmid DNA or PCR products for cloning was performed using commercially available kits from Qiagen or Zymo Research. All the codons of the heterologous genes were optimized using the online tool COOL [48]. All chemicals were reagent grade and obtained from Sigma-Aldrich (St. Louis, MO) or Fisher Scientific (Pittsburgh, PA) unless indicated otherwise. CTFUD-rich medium [49] was used for routine strain maintenance, and MTC-5 defined medium [8] was used for fermentation as indicated. *C. thermocellum* transformation was performed as previously described [49].

Serum bottle batch cultures were incubated at 55 °C and shaken at 180 rpm. Serum bottles were purged with N_2_ and sealed with butyl rubber stoppers to prevent gas exchange. In bottle fermentations, pH was controlled with 40 mM MOPS buffer. MTC-5 medium with 20 g/L cellobiose was used for fermentation. For the *n-*butanol pathway combination experiment, all the strains were cultured in serum bottles and with MTC-5 medium for 7 days. Bioreactor fermentations were carried out in 1.5-L (1-L working volume) Sartorius Biostat A-plus Sartorius Stedim (Sartorius Stedim, Bohemia, NY) bioreactors in modified MTC-5 medium (no MOPS buffer and with 2 g/L urea as the nitrogen source), with the temperature maintained at 55 °C and stirred at 150 rpm. 50 g/L Avicel PH105 was used as the carbon source. The pH was controlled at 7.0 with a Mettler Toledo pH probe (Columbus, OH) and addition of 8 M KOH. The vitamin supplementation solution contained pyridoxamine dihydrochloride 0.04 g/L, PABA 0.008 g/L, d-biotin 0.004 g/L, vitamin B-12 0.004 g/L. The vitamin supplementation solution was filter sterilized and added after autoclaving the bioreactor. The bioreactor was inoculated with 5% v/v transfer of a fresh seed culture grown on 5 g/L Avicel PH105 in MTC-5 (0.5% v/v). The headspace of the bioreactor was flushed with an anaerobic gas mixture (80% N_2_ and 20% CO_2_) prior to inoculation. Thiamphenicol (dissolved in dimethyl sulfoxide) was added to the medium to a final concentration of 15 μg/mL as a selective agent to maintain the plasmid. 16S rRNA gene sequences of cell pellets from each fermentation were used to verify culture purity.

### Analytical methods

Acetate, formate, ethanol, glucose, *n-*butanol and residual cellobiose were determined by high-pressure liquid chromatography (HPLC) (Waters, Milford, MA, USA) with refractive index detection using an Aminex HPX-87H column (Bio-Rad, Hercules CA) with a 5 mM sulfuric acid solution as the mobile phase. The carbohydrate content of Avicel present in the fermentation samples was determined by quantitative saccharification (QS) using 72% H_2_SO_4_ (Fisher; Waltham, MA) as described by Sluiter et al. [50]. Acid-hydrolyzed sugars (glucose, xylose and arabinose) were quantified by the same HPLC system.

### Protein purification

Target genes were amplified by PCR with Q5 DNA polymerase (New England Biolabs, Ipswich, MA, USA). The target genes were inserted into plasmid pD861-CH (ATUM, Newark, CA, USA) and tagged with a 5× His6 cassette. The vector was transformed into *E. coli* BL21(DE3) for protein expression. The purification of proteins in *E. coli*, cell preparation and cell-free extract were performed as described previously [51]. Cells were grown aerobically in TB medium at 37 °C with a stirring speed of 225 rpm. When the OD_600_ reached 0.6, 4 mM rhamnose was added to induce expression of the target gene. Cells were then grown aerobically for 4 h before harvesting by centrifugation. Cell pellets were washed with buffer (50 mM Tris–HCl, pH 7.5 and 0.5 mM DTT) and stored at − 80 °C.

Cell pellets were resuspended in lysis buffer (1X BugBuster reagent (EMD Millipore, Darmstadt, Germany) with 0.2 mM dithiothreitol). For aldehyde and alcohol dehydrogenases, all the purification and enzyme assay steps were performed in an anaerobic chamber with less than 5 ppm oxygen. The cells were lysed with Ready-Lyse lysozyme (Epicentre, Madison, WI, USA), and DNase I (New England Biolabs, Ipswich, MA, USA) was added to reduce the viscosity. After incubation for 30 min at room temperature, the resulting solution was centrifuged at 10,000×*g* for 5 min. The supernatant was used as cell-free extract for enzyme assays or protein purification.

All purification steps were performed at room temperature as described previously [52]. His-tag affinity spin columns (His SpinTrap, GE Healthcare BioSciences, Pittsburgh, PA, USA) were used to purify the protein. The column was first equilibrated with binding buffer (50 mM sodium phosphate, 500 mM NaCl, 20 mM imidazole, pH 7.5). Cell-free extracts (in 50 mM sodium phosphate, 500 mM NaCl, 20 mM imidazole, pH 7.5) were applied to the column, and then the column was washed twice with wash buffer (50 mM sodium phosphate, 500 mM NaCl, 50 mM imidazole, 20% ethanol, pH 7.5). The His-tagged protein was eluted with elution buffer (50 mM sodium phosphate, 500 mM NaCl, 500 mM imidazole, pH 7.5).

### Enzyme assays

Enzymes were assayed at 55 °C with a BioTek PowerWave XS plate reader (BioTek Instruments Inc., Winooski, VT, USA). The reaction volume was 0.2 ml, with a 0.5 cm path length. All enzyme activities are expressed as μmol of product per minute per mg of cell extract protein. All enzyme assays used 100 mM MOPS-KOH (pH 7.5 at 22 °C) and 0.3 mM NADH or NADPH (as needed).

Thiolase (EC: 2.3.1.9) was assayed with 100 to 4000 μM acetyl-CoA at a final enzyme concentration of 1.2 µg/mL, with the coupled *β*-hydroxybutyryl-CoA dehydrogenase enzyme at 50 µg/mL. *β*-Hydroxybutyryl-CoA dehydrogenase (EC: 1.1.1.35) was assayed with 50 to 500 μM acetoacetyl-CoA at a final enzyme concentration of 0.2 µg/mL. Crotonase (EC: 4.2.1.55) was assayed with 25 to 1000 μM 3-hydroxybutyryl-CoA at a final enzyme concentration of 0.5 µg/mL, with the coupled trans-2-enoyl-CoA reductase enzyme at 100 µg/mL. Trans-2-enoyl-CoA reductase (EC: 1.3.1.44) was assayed with 25 to 750 μM crotonyl-CoA at a final enzyme concentration of 2 µg/ml. Acetaldehyde (EC: 1.2.1.10)/butyraldehyde dehydrogenase (EC: 1.2.1.57) was assayed with 50 to 500 μM acetyl-CoA or 50 to 500 μM butyryl-CoA at a final enzymes concentration of 15 µg/mL for AdhE2 and 5 µg/mL for Bad. Ethanol/butanol dehydrogenase (EC: 1.1.1.1) was assayed with 0.5 to 20 mM acetaldehyde or 0.5 to 20 mM butyraldehyde at a final enzyme concentration of 15 µg/mL for AdhE2 and 2 µg/mL for Bad. The formation NADH or NADPH was followed by photometric observation at 340 nm (ε = 6.2 mM^−1^ cm^−1^) in a BioTek PowerWave XS plate reader (BioTek Instruments Inc., Winooski, VT, USA). The protein concentration was determined using the Bradford protein reagent with bovine serum albumin as the standard (Bio-Rad, Hercules, CA).

### Cofactor specificity reversal library construction and screening

The library was constructed using the CSR-SALAD online tool, and reports from that tool are presented in Additional file 2: Figure S2 [39]. For 3-hydroxybutyryl-CoA dehydrogenase, crystal structure from *Clostridium butyricum* was used as the template (PDB: 4KUG). Three suggested residues were transferred to the 3-hydroxybutyryl-CoA dehydrogenase of *T. thermosaccharolyticum*. The libraries of the three suggested residues were constructed, respectively, and enzyme assay was used to screen the variants. The number of colonies screened was fivefold larger than the library size, to ensure complete coverage (e.g., for the D31 position of Tt_Hbd, the CSR-SALAD suggested substitution codon is RNC, which codes for 8 different variants, so 40 colonies were selected for screening). The best mutations at each position were combined to generate the final variant protein. The same process was applied for the trans-2-enoyl-CoA reductase of *Spirochaeta thermophila* and crystal structure from *Treponema denticola* was used as the template (PDB: 4FBG).

### Whole-genome sequencing for strain confirmation

Genome resequencing was performed as previously described [53]. Briefly, genomic DNA was submitted to the Joint Genome Institute (JGI) for sequencing with an Illumina MiSeq instrument. Unamplified libraries were generated using a modified version of Illumina’s standard protocol. 100 ng of DNA was sheared to 500 bp using a focused ultrasonicator (Covaris). The sheared DNA fragments were size selected using SPRI beads (Beckman Coulter). The selected fragments were then end repaired, A tailed, and ligated to Illumina compatible adapters (IDT, Inc) using KAPA’s Illumina library creation kit (KAPA Biosystems). Libraries were quantified using KAPA Biosystems next-generation sequencing library qPCR kit and run on a Roche LightCycler 480 real-time PCR instrument. The quantified libraries were then multiplexed into pools for sequencing. The pools were loaded and sequenced on the Illumina MiSeq sequencing platform utilizing a MiSeq Reagent Kit v2 (300 cycle) following a 2 × 150 indexed run recipe. Paired-end reads were generated, with an average read length of 150 bp and paired distance of 500 bp. Raw data were analyzed using CLC Genomics Workbench, version 11 (Qiagen, USA). Reads were mapped to the reference genome (NC_017992). Mapping was improved by two rounds of local realignment. Raw data are available from the JGI Sequence Read Archive (https://www.ncbi.nlm.nih.gov/sra).

## Additional files

**Additional file 1.** Comparison of *T. thermosaccharolyticum* thiolase wt and M2 mutant enzyme activity with different concentrations of CoA.

**Additional file 2.** Report generated by the CSR-SALAD algorithm for the Hbd (4KUG) and Ter (4FBG) proteins suggesting mutations that might change cofactor specificity from NADH to NADPH.

## Data Availability

All data generated or analyzed during this study are included in the published article and its additional files. DNA sequences and resequencing results are available from GenBank via their accession numbers (see Tables 6 and 7).
