# Supplementary figures and images for: Metabolic engineering of Clostridium thermocellum for n-butanol production from cellulose
Source: Biotechnol Biofuels. 2019 Jul 23;12:186. doi: 10.1186/s13068-019-1524-6 (PMC6652007; doi:10.1186/s13068-019-1524-6)

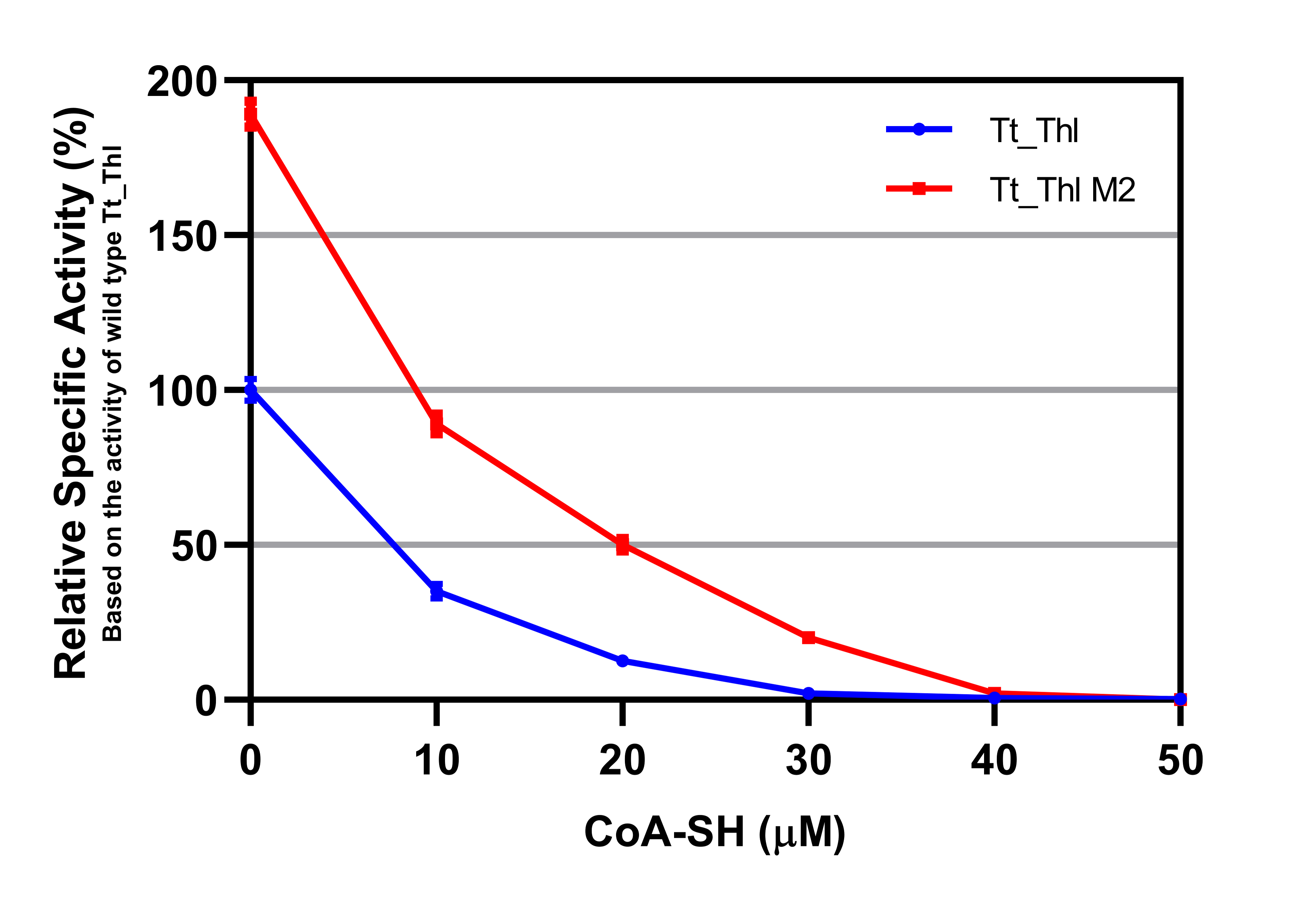

Supplement: Supplementary file 1 — Additional file 1. Comparison of T. thermosaccharolyticum thiolase wt and M2 mutant enzyme activity with different concentrations of CoA. [file 13068_2019_1524_MOESM1_ESM.tif]
