# Supplementary material for: Metabolic engineering of Clostridium thermocellum for n-butanol production from cellulose
Source: Biotechnol Biofuels. 2019 Jul 23;12:186. doi: 10.1186/s13068-019-1524-6 (PMC6652007; doi:10.1186/s13068-019-1524-6)
Supplement: Supplementary file 2 — Additional file 2. Report generated by the CSR-SALAD algorithm for the Hbd (4KUG) and Ter (4FBG) proteins suggesting mutations that might change cofactor specificity from NADH to NADPH. [file 13068_2019_1524_MOESM2_ESM.pdf]

CSR-SALAD

cofactor specificity reversal
structural analysis & library design

Input About

Analysis Results for 4KUG

Residue	Type	Codon	AAs
ASP 31	Edge	RNC	ADGINSTV
ILE 32	Face	ANA	IKRT
PHE 36	Simple	WHC	FINSTY

Suggested library size: 192

The following residues should be targeted first for activity-recovery by site-saturation mutagenesis:

Medium priority:
LEU 7
ARG 30
ALA 88
ILE 94
ILE 98

Low priority:
GLU 86

Analysis Results for 4FBG

Residue	Type	Codon	AAs
SER 55	Bidentate	RSC	AGST
ASN 56	Pyrophosphate	ARC	NS
SER 78	Simple	RSC	AGST
GLU 80	Nonsimple	DMK	ADEKNSTY*

Suggested library size: 384

The following residues should be targeted first for activity-recovery by site-saturation mutagenesis:

Medium priority:
PHE 79
GLY 115
ASP 116
ALA 117
PHE 118
LEU 144
ALA 145
SER 146
VAL 148

Low priority:
THR 90
ASN 96
THR 112
ASP 114
ASP 116
ASP 205
